# Effect of Aging on Tendon Biology, Biomechanics and Implications for Treatment Approaches

**DOI:** 10.3390/ijms242015183

**Published:** 2023-10-14

**Authors:** Ka Yu Carissa Kwan, Ka Wai Kerry Ng, Ying Rao, Chenxian Zhu, Shengcai Qi, Rocky S. Tuan, Dai Fei Elmer Ker, Dan Michelle Wang

**Affiliations:** 1 School of Biomedical Sciences, Faculty of Medicine, The Chinese University of Hong Kong, Hong Kong SAR, China; carissakwan@link.cuhk.edu.hk (K.Y.C.K.); ngkerry1997@gmail.com (K.W.K.N.); yingrao@link.cuhk.edu.hk (Y.R.); e0664588@u.nus.edu (C.Z.); tuanr@cuhk.edu.hk (R.S.T.); elmerker@cuhk.edu.hk (D.F.E.K.); 2 Institute for Tissue Engineering and Regenerative Medicine, The Chinese University of Hong Kong, Hong Kong SAR, China; 3 Department of Prosthodontics, Shanghai Stomatological Hospital, Fudan University, Shanghai 200040, China; qishengcai@fudan.edu.cn; 4 Center for Neuromusculoskeletal Restorative Medicine, Hong Kong Science Park, Hong Kong SAR, China; 5 Department of Orthopaedics and Traumatology, Faculty of Medicine, The Chinese University of Hong Kong, Hong Kong SAR, China; 6 Ministry of Education Key Laboratory for Regenerative Medicine, School of Biomedical Sciences, The Chinese University of Hong Kong, Hong Kong SAR, China

**Keywords:** tendon aging and degeneration, tendinopathy, tendon stem/progenitor cells, extracellular matrix, biomechanical properties, tendon healing, treatment and therapy

## Abstract

Tendon aging is associated with an increasing prevalence of tendon injuries and/or chronic tendon diseases, such as tendinopathy, which affects approximately 25% of the adult population. Aged tendons are often characterized by a reduction in the number and functionality of tendon stem/progenitor cells (TSPCs), fragmented or disorganized collagen bundles, and an increased deposition of glycosaminoglycans (GAGs), leading to pain, inflammation, and impaired mobility. Although the exact pathology is unknown, overuse and microtrauma from aging are thought to be major causative factors. Due to the hypovascular and hypocellular nature of the tendon microenvironment, healing of aged tendons and related injuries is difficult using current pain/inflammation and surgical management techniques. Therefore, there is a need for novel therapies, specifically cellular therapy such as cell rejuvenation, due to the decreased regenerative capacity during aging. To augment the therapeutic strategies for treating tendon-aging-associated diseases and injuries, a comprehensive understanding of tendon aging pathology is needed. This review summarizes age-related tendon changes, including cell behaviors, extracellular matrix (ECM) composition, biomechanical properties and healing capacity. Additionally, the impact of conventional treatments (diet, exercise, and surgery) is discussed, and recent advanced strategies (cell rejuvenation) are highlighted to address aged tendon healing. This review underscores the molecular and cellular linkages between aged tendon biomechanical properties and the healing response, and provides an overview of current and novel strategies for treating aged tendons. Understanding the underlying rationale for future basic and translational studies of tendon aging is crucial to the development of advanced therapeutics for tendon regeneration.

## 1. Introduction

The worldwide increase in life expectancy is expected to exacerbate aging-related declines in tissues such as tendons, highlighting the need for a better understanding of the inherent biological changes and their impact on musculoskeletal function. This has driven the development of advanced therapeutics to alleviate their socioeconomic impact and burden. Global aging-associated injuries and diseases account for 23% of the total disease burden (574 million of the 2490 million disability-adjusted life years (DALYs)) in people aged 60 years or older, with musculoskeletal disorders as the fourth leading contributing factor [1,2]. Aging induces degeneration, dysfunctional regenerative capacity, and eventually, functional impairment of body systems [3]. Specifically, aging is a key predisposing factor for tendinopathy, one of the most common tendon disorders which accounts for 50% of reported musculoskeletal injuries presented for medical interventions [4,5]. The onset of microtrauma and subclinical inflammation in aged and/or overused tendons gradually leads to the development of pain, swelling and physical hindrance throughout our lifetime [6]. Tendon aging can substantially impact cell behavior, extracellular matrix (ECM) composition, biomechanical features, and healing capacity. In response to tendon-aging-related symptoms, current clinical interventions are predominantly conservative, such as pain/inflammation management and minimally invasive surgery [7]. Recently, more advanced therapies, such as aged tendon cell rejuvenation [8,9], have been shown to have the potential to reverse the detrimental effects of aging and achieve the ambitious goal of functional tendon regeneration. In this study, we review recent work on age-related tendon changes in terms of cells, ECM, biomechanical properties, and healing capacities. We also discuss conventional (diet, exercise, and surgery) and advanced (cell rejuvenation) treatments for aged tendon repair and regeneration. Understanding the biology of tendon aging and the rationale of therapeutic approaches is crucial for the development of effective interventions for this significant health concern.

## 2. Biology of Tendon Aging

Aging has a significant impact on tendons, affecting both their constituent cells and ECM. As a connective tissue whose primary function is to transmit muscle-generated contractile force to bone, the collective actions of tendon cells are important for maintaining the homeostatic state of the body. However, undesired molecular changes to this ECM-rich tissue may negatively influence its mechanical attributes [2,6].

### 2.1. Tendon Cellular Changes during Aging

Tendons are comprised of distinct cell populations of cells, including tendon stem/progenitor cells (TSPCs), tenocytes, tenoblasts, and vascular cells [10]. TSPCs are precursors of tenocytes and tenoblasts (resident tendon cells) and reside within the unique tendon niche. TSPCs share stem cell characteristics (clonogenicity, multipotency, self-renewal), however, are distinct from bone-marrow-derived stem cells (BMSCs). In addition to cell surface markers indicative of stemness, e.g., stem cell antigen-1 (Sca-1) and CD44, TSPCs highly express the tendon-specific markers type I collagen (COL1A1), Scleraxis (SCX), and tenomodulin (TNMD) and lack the BMSC surface receptor CD18 [11]. Tenocytes and tenoblasts are collectively known as tendon cells. As no specific markers are identified for the two cell types, such distinction is based on the cell shape anchored on the ECM. Tenocytes are spindle-shaped tendon-specific fibroblasts; tenoblasts are round with oval nuclei. Tenoblasts are also known to be the active form of tenocytes that orchestrate the intrinsic healing stage during aging [12,13]. Cellularity, morphology and behavior of tendon cells and their progenitor cells are influenced by aging. These age-related changes are discussed in the following sections and summarized in Table 1.

#### 2.1.1. Tendon Cellularity and Cell Morphological Properties during Aging

Most studies have shown that as the postnatal tendon matures, the number of tendon cells rapidly declines, while the ECM expands substantially. During aging, the number of tendon cells decreases even further, leading to a reduction in the already low populations of TSPCs [14,15]. However, there are exceptions to this pattern. For example, in horse superficial digital flexor tendons (SDFTs), no decrease in cell numbers or DNA content has been reported [16].

Distinct cell morphological changes have been observed in many different studies, with a general trend showing that young tendon fibroblasts contain numerous well-defined actin fibers oriented along the longitudinal axis of elongated cells, while aged tendon fibroblasts are more rounded with a poorly organized actin cytoskeleton, as well as differential localization of key focal adhesion (FA) proteins [15,17]. However, a recent study using atomic force microscopy (AFM) and two-photon excited fluorescence (TPEF) microscopy revealed that TSPCs isolated from young patient donors contained only a few dominant fibers that spanned the longitudinal axis of the cell, and the actin fibers did not strictly align in parallel. In contrast, aged TSPCs displayed a more uniformly distributed actin cytoskeleton. Compared with aged TSPCs, young TSPCs also exhibited larger fiber-to-fiber distances, especially for nuclear-spanning stress fibers [18]. Similarly, increased viscoelastic properties of aged TSPCs were observed via quartz thickness shear mode (TSM) resonators [19]. This phenotype of irregular cell shape and a more densely packed actin fiber network in aged TSPCs may contribute to the increased cell stiffness and viscoelasticity during aging, which can potentially be used as indicators for tendon cell aging.

#### 2.1.2. Cell Motility, Metabolism and Self-Renewal Capacity during Aging

The ability of cells to migrate toward an injury site, along with an active metabolic state, is essential for their participation in wound healing. In aged individuals, tendon cells exhibit a decrease in motility, such as adhesion and migration, which is speculated to contribute to the decreased tendon healing capacity [15,17]. Mechanistically, decreased tendon cell motility is closely related to changes in actin fiber organization and FAs. FA components, such as focal adhesion kinase (FAK), paxillin and talin, are important players that regulate cell behavior [17,20,21]. Arnesen et al. reported that these FA proteins were mostly located at the cell surface of young tendon fibroblast but aggregated around the nucleus in aged tendon fibroblast. Since FAs are critical for generation of the traction force necessary for cell migration, the absence of FAs at the cell surface may influence the adhesion and migration efficacy in cells of aged mammalian species [17]. Additionally, the overall metabolic activity of tendon cells also decreases with aging. A classic study showed that rabbit Achilles tendon tissue slices had high levels of aerobic glycolysis as the main energy source during the first three months of extrauterine life, which then gradually decreases. In comparison, anaerobic glycolysis remained relatively stable throughout the lifespan [22]. Such decreases in migration ability and altered levels of metabolic activity may explain the decreased tendon healing in aged individuals.

In addition to cell motility and the metabolic state, there must be active TSPCs that can generate sufficient numbers of tissue-resident cells (tenocytes) to replenish those lost to injury. Within this context, altered TSPC self-renewal capacity during aging has been implicated in age-related tendon disorders and diseases [8]. Tendon fibroblasts isolated from aged donors (mice) have doubling times up to approximately three times longer compared to tendon fibroblasts isolated from young donors. [17] TSPCs also exhibit decreased proliferation rates, delayed cell cycle progression, and altered cell fate patterns. As aging progresses, TSPCs lose their ability to self-renew and sustain their population, eventually reaching depletion. This process can be further accelerated such as early entry into senescence upon activation of cellular defense mechanisms [23]. Kohler et al. reported that aged TSPCs had premature entry into cell senescence, which reflects an intriguing transcriptomic shift, where the most differentially expressed genes were associated with regulation of cell adhesion, migration, actin cytoskeleton, and dysregulated cell–matrix interactions [23]. As such, the reported changes in aged TSPCs may lead to disruption of tissue homeostasis.

Mechanistically, signaling molecules such as Rho-associated protein kinase (ROCK) have been widely explored in TSPC aging and degeneration. ROCK plays a critical role in cellular processes affected by aging, including cell morphology, mitosis, motility and senescence [24]. Treating aged TSPCs with a ROCK inhibitor (e.g., inhibiting ROCK1 and 2) reversed some of these age-related changes, such as acquiring a more spindle-like cell shape and reducing stiffness [18]. ROCK1 is also the direct target of microRNA (miR)-135a, which is significantly downregulated in aged TSPCs [24]. Furthermore, growth-arrest- and DNA-damage-inducible gene 153 (GADD153) is expressed at very low levels in growing cells but is markedly induced in response to a variety of cellular stresses, including excessive protein misfolding. In aged tendon fibroblasts, GADD153 expression was substantially higher than in young tendon fibroblasts, suggesting that a higher level of unfolded or misfolded proteins was present in aged tendon fibroblasts [17]. Other possible mechanisms, such as those involving Cbp/p300-interacting transactivator 2 (CITED2, a multi-stimuli responsive transactivator), CD44 (a matrix assembling and organizing protein implicated in tendon healing), TNMD (a well-known gene marker for the tendon and ligament lineage which plays a regulating role on cell proliferation and adhesion), peptidyl-prolyl cis-trans isomerase NIMA-interacting 1 (PIN1, a highly conserved peptidyl-prolyl isomerase (PPI) with anti-aging roles in TSPCs), aquaporin 1 (AQP1, a member of the small water-transporting membrane protein family), forkhead box (FOX) P1 (FOXP1, belongs to subfamily P of the FOX transcription factor family) and connective tissue growth factor (CTGF, a cysteine-rich secretory protein belonging to the cellular communication network family), have also been recently discovered to be involved in TSPC senescence and repair capacity [14,25,26,27,28,29]. While the underlying mechanisms for the reduced self-renewal capacity during aging remain unknown, continued investigations should help to identify novel tendon-specific therapeutic targets to enhance the regenerative capacities of adult and aged tendons.

#### 2.1.3. Cell Differentiation and ECM-Secreting Potential during Aging

To ensure effective tissue homeostasis, tenogenesis, along with tendon ECM production and secretion, must be sustained. Generally, protein synthesis is reduced in aged tenocytes, likely related to changes in cytoplasmic organelles, such as the rough endoplasmic reticulum and mitochondria [30], as well as dysregulated cell–cell interactions [31]. Sugiyama et al. reported that in mouse flexor tendons, the mRNA expression levels of collagens (type I and type III) and tenogenic markers (Mohawk homeobox (MKX), SCX and TNMD) declined substantially with aging [32]. Similarly, transmission electron microscopy showed that in comparison to adult rat fibrocartilage cells at the bone–tendon junction, aged cells had shorter and fewer cytoplasmic processes (and thus, a reduced Golgi network and protein synthesis capacity), fewer granular endoplasmic reticulum cisterns and fewer mitochondria [30]. Conversely, Thorpe et al. showed no substantial decline in mRNA or protein levels of matrix proteins and degradative enzymes in horse SDFT specimens from 3–30 years of age [16].

The role of TSPCs in tendon maintenance and regeneration has recently received increased attention. Many studies have shown that young and aged TSPCs stain positively for three stem cell markers: nucleostemin, octamer-binding transcription factor 4 (OCT4), and stage-specific embryonic antigen-4 (SSEA4). These characteristics indicate that the aged TSPC population may retain the ability for multipotency [8,33]. However, there are also conflicting results that aged TSPCs have reduced stemness, trilineage differentiation potential, and ECM synthesis [3,29,33,34,35]. Interestingly, a recent study showed that compared with young TSPCs, aged TSPCs were less competent and formed thin and fragile three-dimensional tendon organoids, largely due to increased cell apoptosis and senescence as well as deficient matrix production [36]. Recent discoveries suggest that these age-related changes in TSPCs are related to epigenetic modifications, decreased levels of growth factors and hormone deficits [8,29]. Thus, understanding the methylation status of aged TSPCs and/or alteration of their surrounding microenvironment [8,29,37,38], may help to reveal the mechanism of TSPC aging and restore their regenerative potential.

**Table 1:** Age-related changes in cellular characteristics and behavioral properties.

	Age-Related Changes	Description	Reference
**Cell physical property**	Cellularity	Cell numbers in tendons are reduced with aging.	[3,32,39,40,41,42,43]
		DNA content does not decrease with aging in horse SDFTs.	[16]
		The TSPC pool is exhausted with tendon aging and degeneration.	[23]
		Possible mechanisms of TSPC aging and degeneration: ROCK pathway, miR-135a, Pin1, and TNMD.	[18,24,25,26]
	Cell morphology	Young TSPCs can show a spindle-like cell shape; aged TSPCs exhibit a star-like flattened appearance.	[18,19,23]
		Young tenocytes have a rounder cell shape, but aged tenocytes have a thinner, more elongated phenotype.	[32,40]
		Aged tenocytes show a higher nucleus-to-cytoplasm ratio and elongated nucleus compared with young tenocytes.	[32,44]
		Tenoblasts become longer and more slender with aging.	[45]
	Cytoskeletal organization	In young TSPCs, only a few dominant fibers span the longitudinal axis of the cells and do not align strictly in parallel. Aged TSPCs show a well-structured actin cytoskeleton and a smoother surface without prominent fibers.	[18,39]
		Aged tendon fibroblasts have fewer stress fibers, which are only present close to the cell periphery.	[17]
	Stiffness	Aged TSPCs are generally stiffer than young TSPCs.	[18,19]
**Cell behaviors**	Cell adhesion and migration	Slower actin dynamics might contribute to the decreasing migratory capacity of TSPCs during aging.	[14]
		Young tendon fibroblast adhered 30% more efficiently and formed stronger attachment than aged tendon fibroblast. FA proteins displayed different localization in tendon fibroblast with aging.	[17]
	Metabolic activity	There is a reduction in the organelles participating in protein synthesis, e.g., rough endoplasmic reticulum (ER), mitochondria.	[46]
		In rabbit Achilles tendon tissue slices, aerobic glycolysis gradually decreases from the first three months and cellular respiration completely ceases at three years of age, while anaerobic glycolysis is maintained at a similar activity level throughout the whole tendon lifespan.	[22]
		There is a higher GADD153 expression (ER stress-related) in aged tendon fibroblast than in young tendon fibroblast.	[17]
	Cell–cell interactions	Aged TSPCs cell–cell interactions are limited by the downregulation of the ephrins (Eph) receptors EphA4, EphB2, EphB4 and ligand EFNB1.	[31]
	Proliferation (self-renewal) capacity	The proliferation of TSPC is reduced with aging. Cell cycle progression was arrested at G2/M phase in senescent TSPC. These phenomena are associated with the downregulation of cellular senescence-inhibited gene (CSIG) and upregulation of p27.	[14,34]
		The doubling time of aged tendon fibroblast is about three times slower than that of young tendon fibroblast.	[17]
	Differentiation	Aged TSPCs showed reduced stemness, while other groups have shown that young and aged TSPCs were stained positively for three stem cell markers, indicating that this population retains their stemness regardless of age.	[14,34,35]
		In TSPCs, CD44 was upregulated with aging, but CITED2, FOXP1 and Pin1 were downregulated.	[14,26,28]
		The tenogenic differentiation capacity of TSPCs significantly decreases with aging. The p16/miR-217/early growth response factor 1 (EGR1) pathway is involved in the age-related tenogenic differentiation of TSPCs.	[47]
		In an RNA-sequencing analysis, the expression of 325 transcribed elements was significantly distinct between old and young tendons.	[48]
		The mRNA expression levels of collagens and tenogenic markers significantly declined with aging, but contradictory views were mentioned by other authors.	[16,32,41]

### 2.2. Biology of Cellular Tendon ECM during Aging

**Figure 1:**
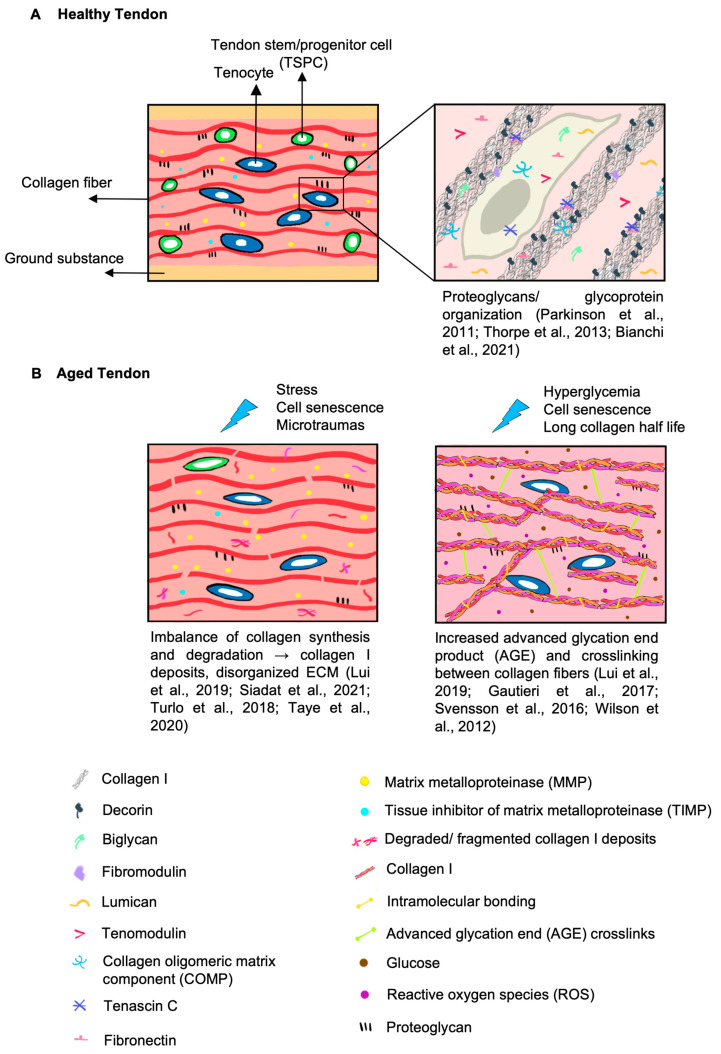
Tendon ECM compositional and structural changes under healthy and aged states. Upper right: [50,51,52] Left: [3,40,53,54] Right: [3,55,56,57]. (**A**) Cellular and extracellular components in healthy tendons. Collagen-secreting tenocytes and their progenitor cells, TSPCs, are located between collagen fibers and crucial for maintaining their anisotropic arrangement and tensile strength [58,59]. Collagen fibers are organized in a parallel and homogenous manner [52,58] and interspersed within other ECM components [52,58]. Matrix metalloproteinase (MMPs) and tissue inhibitor of metalloproteinase (TIMPs) coordinate with PGs to modulate collagen fibrillogenesis, cell/growth factor interactions, matrix assembly and fibril sliding during musculoskeletal movement [49,60,61]. Adapted from [50,52]. (**B**) Age-related changes in tendon cellular and ECM components. TSPC or tenocyte dysfunctionality [47,62] as well as an increased MMP/TIMP ratio [3] contribute to collagen fragmentation and results in a disorganized ECM [53,63]. In addition to intramolecular bonding (in yellow) between the collagen triple helix, intermolecular bonding or advanced glycation end product (AGE) related crosslinks (in green) increase during aging [56]. AGE formation can be further accelerated by hyperglycemia (in brown) and increased levels of reactive oxygen species (ROS) (in purple) (induced by cell senescence) [64,65]. Alterations of PGs and GPs in aging tendons are inconclusive [3,51,53,66,67]. Adapted from [52,57].

The ECM is the noncellular three-dimensional meshwork within tendons that is a part of the tendon microenvironment and responsible for its mechanical functions. It is composed mainly of collagens and non-collagenous macromolecules, including proteoglycans (PGs), glycoproteins (GPs), and glycoconjugates [49,50]. The ECM is vital to a biologically functional tendon by (1) providing biochemical stimuli for tissue development, regeneration and healing, and (2) serving as a physical scaffold to support its biomechanical activity [49,50]. Tendon ECM features, including compositional and structural changes during aging, are illustrated in Figure 1.

#### 2.2.1. Changes in Collagens during Aging

Collagens are the most abundant proteins in the ECM, accounting for 60–85% of tendon dry weight [49,59], and are the primary contributor to the mechanical properties of tendons. As aging progresses, collagen biosynthesis can be reduced by multiple processes including depletion of collagen-secreting tenocytes and its progenitor TSPC, increased degradation (by remodeling enzymes) and post-transcriptional hydroxylation, glycosylation and AGE crosslinking [49,68]. As a result, reduction in collagen fibril size and fragmented and disorganized collagen fibers are commonly found in the aged tendon ECM [49,59].

Collagen type I accounts for 95% of the total collagen mass and is ubiquitous throughout regions of the tendon [69]. As the most abundant protein in the ECM, collagen type I is responsible for tendon tensile strength and viscoelasticity in addition to fibrillar collagen types III and V, which represent the remaining 5% [49,59,70]. Fibrillogenesis begins with the assembly of collagen type I, followed by linear and lateral growth dictated by other collagen types (types III, V, XI, XII and XIV) [71]. In particular, the thinner collagen type III is critical to initial fibril alignment and extensibility and decreases gradually during development [70]. Buckley et al. established the association of collagen type III content with low tensile modulus and collagen alignment. This attribute may explain that tendon aging and injuries mediated by collagen type III, often result in incomplete healing and weakened mechanical function [69]. Extending towards the tendon–bone interface or the enthesis, collagen types I-III are also expressed at the fibrocartilage transitional zone. Resembling both tendon and cartilage features, collagen type II at the fibrocartilage is responsible for withstanding high compressive load whilst collagen types I and III dissipate the bending of collagen fibers at the tendon [72].

TSPC and tenocyte populations are the major cell types that secrete and organize collagen fibers [49,59]. Under the influence of aging, cell senescence (refer to Section 1 and Section 2 for more details) is anticipated to reduce TSPCs’ proliferative and differentiation abilities as well as tenocyte number and functions [47]. In light of this, collagen synthesis levels and ECM organization are expected to be decreased and/or disrupted. However, contradictory results have been reported. For example, in the Achilles tendon of 22- to 25-month-old rats, the mRNA expression of collagen types I and V was reduced, but collagen protein content remained unchanged compared to that of young rats [3,66]. Another study revealed that collagen type VI, which plays a significant role in collagen fibrillogenesis, was continuously expressed in the aged SDFTs of horses [67]. Additionally, Thorpe et al. found no reduction in DNA content, cell number or collagen synthesis in horse SDFTs and hence proposed that tendon aging may not be cell-mediated but an ECM assembly dysfunction [16]. As such, the relative changes in ECM content and cellularity in aged tendons require further investigations to clarify whether aged and/or senescent tendon cells exhibit reduced or perturbed ECM production.

In addition to ECM production and secretion, ECM degradation via multiple remodeling enzymes, including members of the MMPs family and their inhibitors (TIMPs), is another important factor. To properly heal, a fine balance between anabolic and catabolic processes is required for tendons to adapt to external stimuli such as injury or exercise. The MMP family is categorized according to their targeting ECM component: collagenases (MMP-1, MMP-8, MMP-13), which degrade fibrillar collagens. MMP-2 and MMP-9 are gelatinases that degrade smaller collagen fragments deposited and cleave the denatured collagen and type IV collagen [73]. Stromelysines (MMP-3, MMP-10, MMP-11), matrilysins (MMP-7, MMP-26) and the metalloelastase MMP-12 primarily degrade glycoproteins and proteoglycans. Membrane type (MMP-14–17) activates other MMPs. MMP activity is inhibited by forming non-specific complexes with TIMPs (TIMP 1–4) [73,74].

During aging, the combination of stress, cell senescence, and accumulation of tendon micro-ruptures leads to an imbalance between collagen synthesis and breakdown. Aging upregulates collagenase expression and immune cell production such as neutrophils and macrophages [75,76]. For example, MMP-2 and MMP-9 activity is enhanced, while the levels of the MMP-1 inhibitors TIMP-1 and TIMP-2 were substantially reduced in tenocytes, suggesting a higher rate of collagen turnover and ECM remodeling during aging [3,77]. Although the strong increase in MMP activity may indicate active tendon healing, multiple studies have also reported that altered expression of MMPs/TIMPs is associated with chronic tendon degeneration and pathologies [74]. It is reasonable to postulate that the enhanced proteolytic activity of the collagenases (MMP-1 and MMP-13) and gelatinases (MMP-2 and MMP-9) results in the deposition of collagen proteolytic fragments and impairs the highly ordered ECM, which suppresses adequate collagen turnover by aged tenocytes [16,53,78]. Therefore, the precise balance of the MMP and TIMP ratio is critical to steer tendon healing for proper regeneration.

Structural alterations of collagen including changes in fibril size (also indicated by fibril diameter and cross-sectional area) and collagen crosslinking, can impact tendon function. The overall effect of aging on these parameters is as yet inconclusive, as different observations have been reported, such as an increase in fibril size [3] and cross-sectional area [39], a decrease in fibril diameter [79,80], and a shift in fibril size distribution [3,56]. Nevertheless, aging-associated changes in collagen structure are expected to significantly impact tendon biomechanical properties, such as decreased tensile strength, altered viscoelasticity, limited fiber sliding, and increased stiffness that were observed during aging [3]. Specifically, AGEs are formed nonenzymatically between collagen and sugars (e.g., glucose) through lysine residues, which can increase the rigidity of collagen [3,56,81]. The formation of AGEs in collagen type I can diminish collagen–PG binding, weaken cell adhesion and migration, and denature collagen structures. AGEs also upregulate the activity of transglutaminase in tenocytes, which further promotes collagen crosslinking [3]. Other common phenotypes associated with aging, such as senescent cells and hyperglycemia, can also accelerate the process of AGE formation [55]. Under cell senescence, ROS become more prevalent, which in turn increases the AGE oxidation rate and further degrades collagen [15]. In hyperglycemia, elevated basal levels of blood glucose and ribose provide more sugars for crosslinking through lysine residues. Another factor that increases AGE formation is the collagen content. This is because AGE formation accumulates over years, while tendon collagen, which has a long half-life ranging from 2 months to 200 years (based on the measurement of the ratio of d-aspartate to l-aspartate to estimating protein half-life, alongside the assessment of collagenase-generated neoepitopes and cross-linked telopeptide of type I collagen as degradation markers), is not easily degraded, thus allowing more time for AGE formation [55,82]. Overall, the above-mentioned factors increase AGE-mediated collagen crosslinking, which further disrupts ECM homeostasis during tendon aging.

#### 2.2.2. Changes in Noncollagenous ECM during Aging

Although collagen type I is the major tendon ECM component, noncollagenous ECM components, including PGs, glycosaminoglycans (GAGs) and GPs, are interspersed between collagen fibrils and important for tendon functionality and ECM integrity [3,54].

PGs can generally be divided into large aggregating PGs or small leucine-rich proteoglycans (SLRPs), the latter of which include the widely researched ECM components: decorin, biglycan, fibromodulin, and lumican. SLRPs are characterized by leucine-rich repeat (LRR) structures in the core protein and their associated chondroitin sulfate GAG chains, which influence their binding to collagen [83]. The roles of SLRPs in collagen fibrillogenesis, matrix assembly and interaction with growth factors have thus been well recognized [49,60,61,84]. In particular, decorin is the predominant PG (80% of total PG) in the tensile region of the tendon and belongs to class I SLRP along with biglycan [83]. Decorin binds to collagen I via LRR4-6 and has a higher affinity for collagen type I, compared to biglycan which shares a common binding site [61]. As decorin is able to bind to procollagen (collagen monomer) and binds near the C terminus of collagen (near the intermolecular crosslinking), it affects the lateral growth of collagen fibrils and inter-collagen linking during fibril assembly [85]. By virtue of their highly extended conformations, PGs are important for water accumulation [60] to withstand compressive and tensile forces in the tendon and at the enthesis [49,86].

To date, the overall effect of aging on PGs is still inconclusive [3]. In aged horse SDFTs, there was a reduction in fibromodulin, mimecan and asporin protein levels [3,67]. In a supraspinatus tendon aging study of healthy humans, lower levels of total GAG, chondroitin sulfate and dermatan sulfate were reported, but without apparent changes in other GAG-based ECM components [67,87]. However, a recent study using ultrahigh field magnetic resonance imaging (MRI) revealed elevated levels of PG and GAG contents in intrafasicular chondroid-like bodies in aged horse SDFTs [88]. Additionally, as found in aged mouse patellar tendon and horse SDFTs, decorin expression was consistently upregulated with a greater large fibril population [41,80], and in decorin-null mice, tendon fibril structure and alignment were comparable to those observed in mature patellar tendons [41]. Recent studies indicated that the combination of decorin and biglycan knockdown in aged murine patellar tendons led to a greater increase in viscoelastic properties than knockdown of either SLRPs alone [89,90].

The major GPs found in tendons include lubricin, tenascin-C, cartilage oligomeric matrix protein (COMP) and TNMD [51,54,59]. However, few studies have investigated the changes in these specific GPs in aged tendons. Decreased lubricin expression in aged rats was reported to increase the gliding resistance of fascicular sheets and predispose tendons to mechanical stress-related injury [66]. In aged horses, COMP levels decreased in the injury-prone tensional region of SDFTs, but remained unchanged within the compression region of the common digital extensor tendon [91]. Interestingly, a recent study showed that a less well-known matricellular GP, secreted protein acidic and rich in cysteine protein (SPARC), is a collagen-binding GP that involves in collagen fibril assembly and procollagen processing. SPARC also regulates cell–matrix interactions through signaling, adhesion, proliferation, migration and survival [63,92]. An age-related decrease in SPARC can lead to increased lipid deposition and reduced glucose tolerance, which is a common phenotype found in seniors and could potentially be reversed by exercise [93]. In addition, increased content of elastin, a key ECM component that is highly associated with many GPs, with decreased elastic modulus and failure stress were observed in aged rat tail tendons [51,94]. Taken together, these observations suggest that noncollagenous components also play a vital role in tendon integrity, mechanical response, and the tendon microenvironment.

### 2.3. Tendon Biomechanical Properties and Functions during Aging

The primary function of the tendon is force transmission during musculoskeletal movement. One of the hallmarks of tendon aging is the progressive and often irreversible change in its mechanical properties. This is largely attributed to the underlying changes in tendon cellularity, cell function, collagen turnover rate, collagen fibril diameter and alignment, and collagen crosslinking (including glycation-derived crosslinking) [95,96]. Generally, several parameters are used to assess tendon biomechanical properties, such as tensile strength (ultimate strength and yield strength) [96], tensile modulus [75], stiffness [97], and viscoelasticity [98]. Although these parameters are interrelated, they represent different aspects of the tendon biomechanical properties. For example, stiffness is an indicator of the tendency for an element to resist deformation after being subjected to force, while strength measures how much stress can be applied to an element before it deforms permanently or fractures. These different properties can result in tendons being strong and elastic or strong but rigid. Tendon biomechanical changes during aging are summarized in Table 2.

The correlation between tendon aging and biomechanical properties has been widely studied in animals and humans. Conflicting results exist, which may be influenced by the different models, subject age, tendon conditions, and types of tendons investigated [75,99] (details are shown in Table 2). Although there is no clear conclusion about the effect of aging on the mechanical properties of tendons, in general, aging appears to be associated with either an unchanged or reduced modulus, stiffness and strength across multiple studies [3,56,100]. For example, minimal differences were observed in the maximum strength in mice [101] and human subjects [98] or in the stiffness of aged Achilles tendon [102]. Alternatively, a study comparing Achilles tendon properties in older and younger adults suggested that tendon stiffness and elastic modulus decreased in the older group [97]. On the other hand, in a study including 45 elderly (age ≥ 65 years) and 42 young (age 18–40 years) subjects, a markedly stiffer Achilles tendon was observed in the elderly subjects than in the young subjects [103]. Such contrasting observations may be explained using tendon strains as a criterion for comparison. As muscle strength tends to decline with age, a lower force placed on the tendon may lead to reduced tendon strain, confounding interpretations. Therefore, it is important to evaluate strain to a common force when comparing mechanical properties between human age groups [99], for example, matching age-related activity levels or force production capacity. A study by Stenroth et al. compared young and old tendons with similar force production capacity and revealed no age-related changes in tendon stiffness or increased tendon cross-sectional area. However, such reduced tendon mechanical properties may not necessarily be a disadvantage; instead, they could be an adaptation to match the reduced level of muscle performance in seniors, such as low-loading locomotion, to reduce the likelihood of injuries [102].

**Table 2:** Effects of aging on tendon structure and biomechanical properties.

Parameter	Definition and Human Physiological Range	Age-Related Molecular and Structural Changes	Age-Related Biomechanical Changes
**Tensile strength**	The maximum force the tendon can withstand in tension before tear. Achilles [104] 59 ± 18 MPa Patellar [105] 58.7 ± 16.3 MPa	(1) collagen type I, III or IV abundance [106,107,108], collagen crosslinking or nonreducible AGE crosslinks [101,108,109] (2) MMP production [75] (3) extracellular water and PGs [108,110] (4) tendon cross-sectional area and length [63]	Human consistent reduction with aging across multiple tendon types: Achilles tendon [104], patellar tendon [98], anterior tibialis [111] Animal inconclusive could be attributed to differences in species, age, types and conditions of tendons studies generally include the flexor and tail tendon [63,109], Achilles tendon [96,112] and patellar tendon [79]
**Stiffness**	The extent of resistance to elastic deformation in response to the applied force. Achilles [104] 685 ± 262 N/mm Patellar [113] 4434 ± 562 N/mm	(1) collagen—provides elasticity through its high-entropy containing polypeptide chains in the relaxed state [107] (2) AGE crosslink deposits—increases in lysine glycation and inter-collagen bonding stiffen tendons [63,114] (3) elastin—extends but only becomes elastic when hydrated [107] (4) PGs and water influencing a tendon’s elastic recoil [107] (5) increasing fibril radius and tendon cross-section may affect tendon stiffness and functions contradictorily [63,106]	Human inconclusive [97,103,115,116] aged human patellar tendon mainly showed no changes in stiffness [115] Animal inconclusive [108,112,117] stiffness was increased in aged mouse flexor tendon [63] and tibialis anterior and plantaris tendons [118], which could be region dependent
**Tensile modulus**	The slope of the stress–strain curve in the elastic deformation region that measures stiffness [116]. Achilles [119] 822 ± 211 MPa Patellar [98] 660 ± 266 MPa	Similar contributing biological factors as mentioned with additional factors: (1) pyrrole collagen crosslinks—positive correlation with the elastic modulus [120] (2) MMP production increase—associated with tensile modulus reduction [75] (3) modulus change is independent of collagen fibril morphology or force-generating muscle capacity [75,117]	Human inconclusive in Achilles or patellar tendon [97,98,103,116] Animal inconclusive [96,112,121,122,123] Only aged mouse tibialis anterior tendon and flexor tendon have significant increases in the modulus [63,117] while the increase was substantially greater in the proximal region than in the rest of the tendon [117,118]
**Viscoelasticity**	Represented by tendons exhibiting viscous and elastic characteristics when undergoing deformation Decreased dynamic modulus indicates less resistance to strain and a reduced ability to properly transfer force [41].	(1) collagen type I [100], collagen type III [112], and collagen crosslinks [41]—correlate to a greater elastic modulus, fibril volume fraction and stiffness [100] (2) GAG chains have not been shown to directly influence viscoelastic properties [41,112], but the absence of decorin leads to a reduction in the dynamic modulus in aged tendons [41]	Human inconclusive Both reduction in viscoelasticity [110] or no differences [100] in aged patellar tendons were shown Animal reduction in aged mice patellar tendon [38,86]

## 3. Features of Tendon Healing during Aging

Tendon regeneration and healing are challenging due to their poor intrinsic regenerative capacity and the high mechanical demands exerted during functional movement [124,125]. Aging has substantial impacts on tendons at the molecular, cellular and whole-organ levels. Biologically, decreased cellularity, poor cellular function and a degenerated, inflamed microenvironment are typical features of aging tendons [3]. Biomechanically, aging tendons exhibit lower stiffness and tensile strength and may be more susceptible to injuries [99]. These situations further aggravate the challenges in tendon healing, and a tailored therapeutic approach for treating aged tendons is needed. The following section highlights the impact of aging on tendon healing capacity.

### 3.1. General Tendon Healing Phases

**Figure 2:**
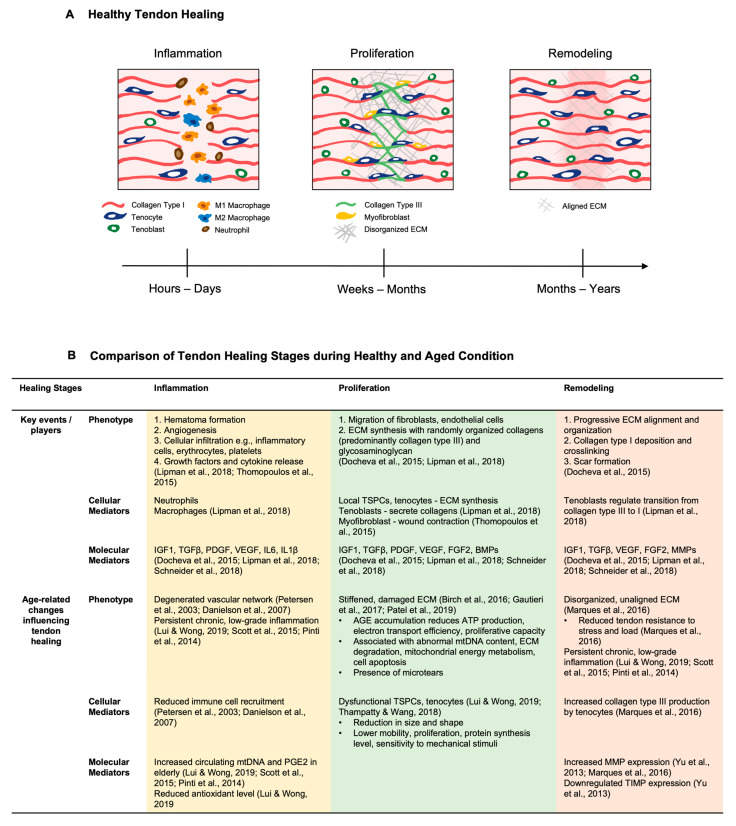
Tendon healing in healthy and aged conditions. (**A**) Schematic diagram illustrating the three general phases of tendon healing. Adapted from [145]. (**B**) Table comparing the tendon healing stages in healthy [126,156,157,158] and aged conditions [3,15,55,77,148,149,150,151,152,154,155].

The tendon healing process is generally divided into three overlapping phases, i.e., inflammation, proliferation, and remodeling, which are summarized in the following sections and in Figure 2A. More detailed reviews can be found in the following articles [126,127,128,129].

The inflammation stage occurs immediately after injury and initiates the healing cascade. During this phase, hematoma formation and cellular infiltration including extrinsic cells (neutrophils, M1 macrophages) and intrinsic cells (such as endotenon and epitenon cells) occur at the injury sites [130]. Subsequently, cytokines including interleukin (IL)-6, IL-1, tumor necrosis factor (TNFα) and growth factors further enhance cell recruitment, proliferation and ECM synthesis. Growth factors relevant to tendon healing include the bone morphogenetic protein (BMP) family, insulin-like growth factor (IGF)-1, epidermal growth factor (EGF), transforming growth factor β (TGFβ), platelet-derived growth factor (PDGF), platelet-derived growth factor (PDGF) and CTGF [130,131]. Angiogenesis is also stimulated at this stage by vascular endothelial growth factor (VEGF), fibroblast growth factor (FGF) and the heparin sulphated (HS)-PG perlecan (involved in FGF2 signaling) [132,133,134]. The crosstalk between growth factors, nitric oxide production and angiogenesis is also modulated by chondroitin sulfate (CS)- and dermatan sulfate (DS)-GAGs [129]. Since collagen synthesis during tendon healing is highly oxygen-dependent, neovascularization at this stage is important for the later formation of capillary networks and ensures sufficient oxygen delivery to the injury site [126,127,135].

Approximately 1–2 weeks after injury, the cell proliferation stage is the dominant response, accompanied by reduced inflammatory responses. The epitenon cells form a thickened layer at the injury site, and tenocytes/tenoblasts are the predominant cell types present at this stage. Their ability to synthesize ECM including collagens, PGs and GPs is upregulated by multiple growth factors, and a randomly organized ECM network is formed [127,136]. Collagen polymerization is assisted by CS- and DS-GAGs and HS-PG on cells that can anchor to the ECM [129]. The migrating tenocytes increase their fibronectin production, which binds to the integrin receptors of the cell surface [137]. Tenascin-C expression also peaks at this point and induces the migratory phenotype of fibroblasts [138]. The synergistic effect provides the scaffold for subsequent adhesion formation [137]. In addition to collagen-building tenocytes, myofibroblasts shrinks the wound size with their extensive cell–matrix adhesions and contractile stress fibers [139]. The transition of fibroblasts to myofibroblasts is driven by versican [140] and hyaluronan (HA) through TGF-β signaling [129]. Yet, its expansion has also been associated with excessive tissue scarring [141].

After 1–2 months, the remodeling stage occurs and can last for several years after the injury. Matrix synthesis will decline, and one remarkable change during the remodeling stage is the progressive ECM alignment and organization. The role of the SLRPs biglycan and decorin in the proliferative remodeling stage has also been investigated. Dunkman et al. reported that the biglycan-null tendon has resulted in significantly lower dynamic modulus 3 weeks post injury [142], along with the upregulation of mRNA expression in a rabbit flexor tendon injury model [143]. Its counterpart decorin adversely affects 3–6 weeks after injury, which could be explained by its role in assembling larger mature collagen fibers [142].

Approximately 10 weeks after injury, the intermediate tissue starts to gradually convert into a functional, scar-like tendon tissue. Although healed, scarred tendons have inferior biomechanical properties compared to the pre-injury tendon [144,145]. These tissues are characterized by the random deposition and orientation of different ECM molecules, contrary to the crimp patterns of collagen fibers in the healthy tendon [146]. Although the scar tissue cross-sectional area increases over the course of healing, and its collagen fiber thickness and tensile strength conventionally increases, the unaligned collagen fibers are immature for force transmission and susceptible to re-injury [146,147].

As such, tendon healing exemplifies the stages and characteristics of general wound healing but is incomplete, resulting in the formation of undesired scar tissue.

### 3.2. Impact of Aging on the Tendon Healing Response

Changes in tendon cellularity and cell function contribute to the age-related decline in tendon healing capacity (Table 2, Figure 2B). Generally, there are fewer tendon cells, including the TSPC pool, in aged tendons than in young tendons. Additionally, cells of aged tendons exhibit lower mobility and proliferation, reduced levels of protein synthesis, and lower sensitivity to mechanical stimuli [3,148] (refer to Section 1 for more details).

During tendon aging, ECM compositional and biomechanical changes, such as an increase in collagen crosslinking and a decrease in tendon strength, lead to a decline in both flexibility and the ability to heal. After injury, tenocytes in aged tendons produce more collagen type III and express abnormally high levels of MMP, especially during the remodeling stage, which further deteriorates the disorganized ECM structure and alignment [149]. Additionally, the accumulation of AGEs in aged tendons reduces adenosine triphosphate (ATP) production, electron transport efficiency and cell proliferative capacity. It is also associated with abnormal mitochondrial DNA content, ECM degradation, mitochondrial energy metabolism and cell apoptosis, together acting as a barrier against cell functionality and tissue regeneration [15,55,150]. Moreover, the degenerated vascular network in aged tendons influences immune cell recruitment from blood vessels [151,152] and ultimately lowers nutritional supply, cell proliferation and ECM synthesis.

Aging tendons are also more susceptible to injuries, including microtears, and associated with a reduced ability to modulate inflammation, which together leads to a chronic low-grade inflammation status termed “inflammaging” [3,15]. Similarly, tendinopathy is a generic term that refers to a type of tendon disorder resulting in pain, swelling, or impaired function, which is also highly associated with tendon overloading, excessive usage, and tendon aging [7]. Certain tendons are more susceptible to damage, including the rotator cuff tendons (particularly the supraspinatus) and biceps brachii tendons in the shoulder, the patellar tendon in the knee, and the Achilles and posterior tibial tendon in the lower leg [153]. Mechanistically, increased immune cell infiltration, cytokine expression, and ECM degradation are implicated during tendon inflammaging [3,7]. Multiple studies also suggest that elderly individuals have lower antioxidant defenses against hypoxia and increased circulating mitochondrial DNA (mtDNA) and prostaglandin E_2_ (PGE2), which substantially contribute to the maintenance of chronic, low-grade inflammation [3,154,155].

As such, inferior cellularity and cell function, an imbalance between ECM synthesis and degradation, decreased tendon biomechanical strength and viscoelasticity, and a reduced capacity to resolve inflammation can synergistically lead to the occurrence of microdamage or sudden tendon ruptures, as well as compromised healing capacity [7].

## 4. Treatment Approaches for Tendon-Aging-Associated Injuries and Diseases

Cellular degeneration, involving TSPCs and tenocytes, plays a significant role in tendon aging, which adversely affects collagen matrix integrity, muscle kinetics and intrinsic healing. Treatment considerations for tendon-aging-associated injuries and diseases follow three main principles: (i) to stimulate cellular activity and protein production, (ii) to reduce harmful biochemical cues/structures and AGE-mediated crosslinks, and (iii) to promote healing, e.g., with the aid of suture techniques/materials and immunological mediators. The following section includes (1) non-invasive treatment approaches such as diet control and exercise; (2) surgical options in elderly patients; and (3) novel strategies focusing on cell rejuvenation.

### 4.1. Diet Suggestions

Diet composition, such as glucose, AGEs, fat, proteins, lipids, magnesium, and fiber, has been shown to influence tendon properties. For example, an AGE-rich diet (e.g., meat, eggs, butter, high fat, fried food) promotes AGE accumulation and has an aging effect on tendons. This is consistent with current evidences that AGE deposition in collagens, such as in diabetic and aged patients, can lead to changes in tendon biomechanical properties and associated with higher incidences of tendon ruptures [159,160,161]. The negative impacts of high fat/lipids or glucose on tendon features have also been explored. A high-fat/glucose diet contributes to nonenzymatic glycation, crosslinking and matrix degradation [95,162], which may deteriorate collagen structure, functionality, elasticity [163,164,165], and impair tendon healing [115,166,167,168]. Conversely, a protein-rich diet, e.g., glycine [169], leucine [170], and supplements, e.g., magnesium [170,171], may exert positive effects, such as increasing ECM synthesis and enhancing the biomechanical characteristics of tendons.

Considering this information, implementing a proper diet may play a positive role in alleviating tendon degeneration and aging. This includes but is not limited to (1) reduced intake of fat-rich products, processed food and sugar; (2) increased consumption of low AGEs, high-leucine and high-glycine foods, such as fish, legumes, low-fat milk products, and vegetables; and (3) proper food processing methods, e.g., avoiding foods cooked at high temperatures and with a low moisture content, which are correlated with higher AGE synthesis levels [95]. The use of olive oil containing the AGE inhibitor aminoguanidine or the antioxidant BHT may completely or partially prevent heat-induced AGE formation. Exposure of meat products to acidic solutions such as lemon juice and vinegar can also reduce AGE formation during cooking [172]. As such, diet control may be beneficial for alleviating the effects of tendon aging and degeneration due to the promotion of tendon-positive effects, such as increased ECM synthesis and enhanced tendon biomechanical attributes.

### 4.2. Physical Activity

As an important intermediary in force transmission for joint movement, tendons are highly responsive to mechanical loading. Exercise can promote tendon healing capacity and reverse the negative impacts of aging in animal models and humans [56,173,174,175,176,177,178], whereas the training period, frequency, and intensity may have various effects on the training results. The mechanism regarding the effects of exercise on tendons has been investigated in multiple studies. An established understanding is that appropriate mechanical stimulation through exercise seems to be more attributed to material adaptations rather than morphological properties [175]. Exercise has also been reported to effectively upregulate cell proliferation/differentiation, growth factor expression and ECM synthesis, as well as to prevent calcification [179]. Specifically, mechanical stimuli cause a physical perturbation to cells via the ECM through interaction with FA, resulting in signal transmission mediated by the cytoskeleton and induced gene expression of the tenogenic marker SCX [180]. Hence, such stimulation can lead to increased collagen fibril density, number, size, turnover rate and accumulation of immature collagen with fewer glycations and crosslinking. On the other hand, excessive mechanical stretching of aging TSPCs was shown to increase non-tenogenic gene expression [34].

The majority of the data suggest that the loading magnitude in particular plays a key role in improving tendon mechanical properties, such as the stiffness and elastic modulus, in contrast to muscle contraction types [56,173,174,175]. Specifically, an effective training intervention should apply a high loading intensity over a long intervention duration, such as over 12 weeks, as suggested by Bohm et al. [175]. In one study, 17 women and 19 men aged 62–70 years were randomly allocated to 12 months of heavy or moderate load resistance training. Despite equal improvements in tendon CSA after training, the use of heavy load training maintained tendon mechanical properties in a manner superior to those with moderate load or no training in older adults [181].

Training may also be effective in diminishing the detrimental effects of aging in tendons, such as ruptures and tendinopathy. Regarding regenerative ability, exercise (especially in older patients with tendon injuries) is essential for faster recovery and higher retainability of normal biomechanical functions. Dressler et al. pointed out that the healing mice patellar tendon could only achieve 20–40% maximum stress of the normal tendon. Thus, shifting the balance towards the normal strut could hinder the repairing tissue from receiving sufficient loading and mechanical stimulation for cell differentiation and reorganization. Through exercise, the introduction of stress and proper posture may reduce the risk of altered joint kinematics [79]. In addition to a carefully planned exercise regimens for aged tendon injuries, a compelling number of studies have also revealed that the addition of partial vascular occlusion (i.e., blood flow restriction (BFR)) during low-load resistance training (20–40% of the 1 repetition maximum) induces beneficial adaptations at the muscular level, such as substantial muscle growth and strength gain comparable to adaptations seen with conventional high-load (HL) training [182,183,184]. Centner et al.’s recent work showed that low-load BFR can also increase Achilles tendon mechanical and morphological properties to a similar extent as conventional HL resistance training. This is of particular importance for individuals who may not tolerate heavy training loads but still aim for improvements in myotendinous function [182]. As such, exercise has beneficial effects for reducing the detrimental effects of aging and degeneration but may need to be tailored to an individual’s specific physical abilities and needs.

### 4.3. Surgical Considerations for Tendon Injury in Elderly Patients

For individuals with severe tendon degeneration, medical interventions such as surgery may be considered. However, there is an ongoing debate regarding surgical treatments for tendon injuries in elderly people. Currently, the initial treatment for tendon injuries is conservative and includes physical therapy, analgesics, corticosteroids, or platelet-rich plasma injections [7,185]. For mid-to-large injuries, various surgical options are available, such as suture- or graft-mediated rotator cuff repair, superior capsule reconstruction, subacromial decompression and reverse shoulder arthroplasty [185]. In elderly patients, non-operative management is the first-line approach, with surgical management being reserved for those who fail a trial of conservative management [185]. This conservative treatment paradigm is intended to lower risks for patients older than 50 years old by avoiding surgery-associated risks such as morbidity or unsatisfactory repair outcomes until deemed necessary [186,187,188]. Indeed, a study evaluating the outcome of a conservative treatment approach for rotator cuff tears in an elderly population showed that treating rotator cuff tears first with conservative therapies and delaying surgery for 3 months was not detrimental to the healing outcome at 3 to 5 years of follow-up. The study included 97 patients (104 shoulders) with a mean age of 68.5 years [186].

In contrast, the literature also shows that surgical repair of tendon injuries can be successfully achieved in elderly patients [185,189,190,191]. For example, elderly patients have been shown to gain as much benefit from arthroscopic rotator cuff repair as their younger peers [192], although aesthetic advantages of arthroscopic techniques are of scarce importance in the majority of middle-aged or elderly patients [193]. In elderly patients, decreased collagen synthesis was shown after short-term unloading similar to young adults, and tendon stiffness and modulus were only marginally affected [194]. Surprisingly, satisfactory results were also reported in severe cases, such as the treatment of infected Achilles tendons with and without secondary tendon reconstruction in elderly patients [195,196,197].

Suture-based repair is commonly used for treating tendon injuries, where suture techniques and suture materials can be important contributors to achieving successful healing outcomes. Sutures can be made from natural or synthetic biomaterials such as silk [198], collagen [199], nylon and polypropylene [200] or poly-lactic and -glycolic acid [201]; thus, sutures serve as a tool to provide temporal mechanical support to facilitate tendon healing [202]. The suture technique is another factor that determines its mechanical support. From conventional Kessler suture techniques to interlockings and modified Kessler loops, an increase in strand numbers and anchor points would greatly increase the mechanical properties of the repaired tendon (e.g., tensile strength and ultimate failure loads) after surgery [203]. Suturing itself usually does not provide a biological contribution for enhanced healing effects. Therefore, the application of sutures solely for the repair of aged tendons that exhibit inferior healing capacity may not be adequate. Accordingly, the development of bioactive sutures for tendon repair has been investigated. These approaches include sutures coated with growth factors (e.g., PDGF, FGF, VEGF, GDF, and neurotrophin [199,204,205,206]); mRNA [207]; ECM proteins such as HA [208]; stem cells [205]; and other bioactive agents such as aloe vera, curcumin, and chitosan [209] and antibacterial agents [210,211,212]. Although not yet well established, such approaches enhancing intrinsic healing capacities may be promising for treating tendon injuries in seniors.

### 4.4. Advanced Cell Rejuvenation Strategies

To address tendon aging, various state-of-the-art strategies have been developed. TSPC’s central role in cellular renewal and subsequent matrix formation has been emphasized in the previous Sections, and undeniably its rejuvenation holds promises to revert age-related changes. In this section, we focus on recent discoveries in TSPC rejuvenation that show great potential for overcoming challenges arising from tendon cellular aging and subclinical, low-grade inflammatory environments.

During aging, compromised cellularity and cell function ultimately influence the tendon’s regenerative capacity. Targeting intrinsic mechanisms, such as membrane and mitochondrial proteins/transcription factors (AQP1, sirtuin 3 (SIRT3), peroxisome proliferator-activated receptor gamma coactivator-1 alpha (PGC1α), and nuclear respiratory factor-1 (NRF1)), Eph receptors, and Rho-kinase, and modulating the extrinsic environment through young ECM coculture, 3D biomaterial scaffold culture and biomechanical stimulation have shown promising efficacy for attenuating cell aging.

TSPC senescence during aging can be rescued through modulation of signaling pathways such as the Janus kinase (JAK)-signal transducer and activator of transcription (STAT), Eph-FAK/p38, and ROCK pathways. Notably, studies have demonstrated that the overexpression of AQP1 restores self-renewal capacity, migration deficits, actin dynamics and tenogenic differentiation in aged TSPCs, and substantially inhibits the phosphorylation of JAK2 and STAT3 [27,35]. Another study reported that the activation of Eph receptors, such as EphA4 and EphB2, normalized the motility and actin turnover of aged TSPCs, but only EphA4 rescued their proliferative ability [31]. A reduction in EphB2 expression in aged mesenchymal stem cells also exhibited a senescence-associated phenotype, emphasizing EphB2′s role in inducing mitochondrial SIRT3 to block aging and oxidative stress [213]. ROCK inhibition has also been a popular topic in which Y27632 inhibition in aged TSPCs rejuvenated cell morphology and stiffness, with a reduced cell volume and more loosely packed actin stress fibers, similar to those seen in young TSPCs [18]. Other anti-senescence targets or pathways, i.e., BMPs, miR-135a, Pin1, CITED2, and the growth hormone (GH)/IGF-1 axis, have been well established [8,24], while p16/miR-217/EGR1, CTGF and inhibitor of nuclear factor kappa-B kinase subunit beta (IKKβ)/nuclear factor kappa-light-chain-enhancer of activated B cells (NF-κB), wingless-type MMTV integration site family, member 5a (WNT5A) signaling, and high mobility group box 1 (HMGB1) have been recently discovered [29,42,47,214,215].

As mentioned in our previous section, oxidative stress or excessive ROS is an important factor leading to cell apoptosis or autophagy. However, a moderate level of ROS has been found to promote cell proliferation and differentiation, which could be induced by hypoxia [216]. Multiple studies have demonstrated the suppressive effect of hypoxia on mitochondrial oxidation, which enhances cell proliferation and upregulates stemness- and tenogenic-related genes [217,218,219]. Nonetheless, hypoxia can also lead to inhibited TSPC multilineage differentiation ability, while excessively low oxygen concentrations impair the TSPC capacity [217]. Therefore, through the precise control of oxygen conditions, it is possible to manipulate TSPC functions. However, the situation during tendon repair is more complicated. Generally, a high oxygen demand is required due to active cell proliferation and protein synthesis during repair. However, oxygen transport to the wound bed is challenging in an avascular tissue such as a tendon, whose environmental condition may contribute to the loss of cells by apoptosis [220]. A study aimed to reactivate mitochondrial function by enhancing the interaction of PGC1α and NRF1 for rotator cuff tendon repair has been reported [221]. They revealed that the promotion of mitochondrial function upregulated various protein synthesis pathways and reduced cellular apoptotic factors.

Apart from altering intrinsic cellular mechanisms, modulating the ECM environment can also be utilized as a cell rejuvenation strategy. During aging, changes in tendon ECM, in terms of biochemical composition and biomechanical properties (refer to Section 3 for more details), may largely influence the regeneration capacity of the resident cells. A previous study showed that a decellularized ECM from young TSPCs enhanced proliferation and tenogenic differentiation, decreased senescence-associated β-galactosidase activity, and preserved stem cell properties of aged TSPCs [222]. These findings suggest that the impaired capacity of aged TSPCs can be rejuvenated via exposure to a young ECM. Another unique approach includes cultivating aged TSPCs in 3D biomaterial scaffolds for cell rejuvenation. For example, to mimic 3D ECM properties, nanofiber hydrogels were developed by using a self-assembling peptide RADA (naturally occurring arginine, alanine and aspartic acid) and incorporating it with an RGD (arginine, glycine, and aspartate) motif. Interestingly, both RADA and RADA/RGD hydrogels not only sustained aged TSPC proliferation and anisotropic alignment but also underwent crosstalk with TSPCs by their ECM modulatory and secretory activities [223].

Biomechanical stimulation is another alternative to modulate the microenvironment to rejuvenate aged TSPCs. Accumulating evidence shows that the biomechanical features of the cell seeding environment can play an indispensable role in manipulating tenogenesis. For example, an in vitro study showed that moderate mechanical stretching of 4% can increase the proliferation, stemness and tenogenic-associated gene expression of TSPCs derived from aged mice. In vivo experiments also revealed that moderate treadmill running by 9-month-old mice reduces tendon degenerative manifestations such as lipid deposition, PG accumulation and tendon calcification [34].

Therefore, the degenerative effect of aging on tendon resident cells can be overcome using cell rejuvenation approaches via stimulatory or inhibitory agents that modulate crucial signaling pathways and reduce oxidative stress, as well as biomechanical conditioning.

## 5. Summary

In conclusion, tendon aging is a natural process that influences tendons at the cellular, molecular, and whole-tissue levels, as well as their biomechanical attributes and regenerative capacity. This process predisposes individuals to an increased risk of injury and disease, even in the absence of symptoms. Therefore, a tailored interdisciplinary approach that combines proper diet, exercise, and novel therapies is needed to promote regenerative properties, provide the appropriate mechanical support, and modulate the inflammatory environment to achieve a favorable healing outcome in age-related tendon injury and disease.

## Data Availability

Not applicable.
